# Metabolic assessment in pure struvite stones formers: is it necessary?

**DOI:** 10.1590/2175-8239-JBN-2020-0106

**Published:** 2021-02-08

**Authors:** Alexandre Danilovic, Thiago Augusto Cunha Ferreira, Samirah Abreu Gomes, Isabela Akemi Wei, Fabio Carvalho Vicentini, Fabio Cesar Miranda Torricelli, Giovanni Scala Marchini, Eduardo Mazzucchi, Miguel Srougi, William Carlos Nahas

**Affiliations:** 1 Universidade de São Paulo, Faculdade de Medicina, Divisão de Urologia, São Paulo, SP, Brasil.; 2 Universidade de São Paulo, Faculdade de Medicina, Departamento de Clínica Médica, Laboratório de Nefrologia Celular, Genética e Molecular, São Paulo, SP, Brasil.

**Keywords:** Nephrectomy, Nephrolithiasis, Risk Factors, Struvite, Nefrectomia, Nefrolitíase, Fatores de risco, Estruvita

## Abstract

**Background and objective:**

Magnesium ammonium phosphate stones (MAP), also known as struvite stones, are associated with urinary infection and impairment of renal unit. The aim of this study is to evaluate the urinary metabolic risk factors for recurrence of renal calculi in patients submitted to nephrectomy due to MAP stones.

**Methods:**

We retrospectively reviewed the charts of patients > 18 years old submitted to total nephrectomy due to pure MAP stones and pure calcium oxalate (CaOx) stones from July 2006 to July 2016. Urinary metabolic parameters were assessed through 24-hour urine exams ≥ 3 months after nephrectomy. Urinary metabolic parameters and new event related to lithiasis were compared.

**Results:**

Twenty-eight and 39 patients were included in MAP and CaOx group, respectively. Abnormalities in 24-hour urine samples were similar between groups. Hypercalciuria occurred in 7.1 and 10.3% of patients in MAP and CaOx group, respectively (*p* = 0.66), whereas hypocitraturia was present in 65.2 and 59.0% of patients with MAP and CaOx group, respectively (*p* = 0.41). No significant difference in new events was found between MAP and CaOx groups (17.9 *vs*. 23.1%, respectively; *p* = 0.60).

**Conclusion:**

A 24-hour urine evaluation should be offered to patients submitted to nephrectomy due to pure MAP stones in order to detect metabolic risk, improve treatment, and prevent stone recurrence.

## Resumo

**Contexto e objetivo:**

Cálculos de fosfato de amônio e magnésio (FAM), também conhecidos como cálculos de estruvita, estão associados à infecção urinária e ao comprometimento da unidade renal. O objetivo deste estudo é avaliar os fatores de risco metabólico-urinários para recorrência de cálculos renais em pacientes submetidos à nefrectomia devido a cálculo de FAM.

**Métodos:**

Revisamos retrospectivamente os prontuários de pacientes > 18 anos submetidos à nefrectomia total devido a cálculos de FAM puro e cálculos de oxalato de cálcio puro (OxCa) de julho de 2006 a julho de 2016. Os parâmetros metabólicos urinários foram avaliados através de exames de urina de 24 horas ≥ 3 meses após a nefrectomia. Os parâmetros metabólicos urinários e um novo evento relacionado à litíase foram comparados.

**Resultados:**

Vinte e oito e 39 pacientes foram incluídos nos grupos FAM e OxCa, respectivamente. As anormalidades em amostras de urina de 24 horas foram similares entre os grupos. A hipercalciúria ocorreu em 7,1 e 10,3% dos pacientes nos grupos FAM e OxCa, respectivamente (*p* = 0,66), enquanto a hipocitratúria esteve presente em 65,2 e 59,0% dos pacientes nos grupos FAM e OxCa, respectivamente (*p* = 0,41). Nenhuma diferença significativa em novos eventos foi encontrada entre os grupos FAM e OxCa (17,9 *vs*. 23,1%, respectivamente; *p* = 0,60).

**Conclusão:**

Uma avaliação de urina de 24 horas deve ser oferecida aos pacientes submetidos à nefrectomia devido a cálculos de FAM puro, a fim de detectar risco metabólico, melhorar o tratamento e prevenir a recorrência de cálculos.

## Introduction

Kidney stone incidence is rising worldwide and it has a recurrence rate of 50% at 5 years after the first episode^1^^,^ 2 ^,^ 3. Magnesium ammonium phosphate stones (MAP), also known as struvite stones, account for 5-15% of cases^4^. These stones are associated with the presence of urease-producing microorganisms, which hydrolyze urea and increase urinary pH, resulting in precipitation of MAP crystals^5^. Struvite stones can occupy the entire renal collecting system, resulting in infectious complications such as xanthogranulomatous pyelonephritis, pyonephrosis, perirenal abscess, and sepsis. In severe cases, these stones can cause renal function loss, associated with recurrent pain and urinary tract infection, and are treated by total nephrectomy^6^.

Nevertheless, other factors can be involved in struvite stone formation since urinary tract infection (UTI) caused by urease-producing bacteria not always produces struvite stones. In fact, other authors showed that the incidence of UTI caused by urease-positive bacteria was around 30% and the incidence of struvite stones was around 15%^7^. On the other hand, patients with pure MAP stone may also present metabolic risk factors for renal calculi formation, such as hypercalciuria, hyperoxaluria, hypocitraturia, and hyperuricosuria, contributing to urolithiasis recurrence^8^.

The present study aimed to evaluate the incidence of urinary metabolic risk factors and its association with renal calculi recurrence after nephrectomy due to pure MAP stones.

## Methods

We performed a retrospective review of electronic medical records of patients > 18 years old submitted to nephrectomy due to pure MAP kidney stones in our institution from July 2006 to July 2016. In order to compare the results, we also reviewed data from patients who underwent nephrectomy due to calcium oxalate (CaOx) stones in the same period. Nephrectomy was indicated by loss of renal function associated with infectious complications such as recurrent urinary tract infection or pyonephrosis in the MAP group or loss of renal function associated with pain in the CaOx group. This study was approved by the Ethics Committee of our institution (research ethics board number: 15394).

Stone composition was determined by chemical analysis. Twenty-four-hour urine samples were collected ≥ 3 months after nephrectomy. The valid samples for inclusion contained urinary creatinine between 1,040 - 2,350 mg/24h for men and 740 - 1,570 mg/24h for women. Exclusion criteria were chronic renal failure stage 4 or 5, urinary tract infection during 24-hour urine collection, presence of contralateral urolithiasis and use of thiazide, citrate, or allopurinol during 24-hour urine collection.

Abnormal 24-hour urinary parameters used were as follows: hypercalciuria > 300 mg/24h of calcium excretion for men and > 250 mg/24h for women; hypocitraturia <320 mg/24h citrate excretion; hypernatriuria > 220 mEq/24h of sodium excretion; hyperoxaluria > 31 mg/24h oxalate excretion; hyperuricosuria > 800 mg/24h of uric acid excretion for men and > 750 mg/24h for women. Comorbidities were classified according to the Charlson comorbidity index^9^ and ASA (American Society of Anesthesiologists) classification^10^. Split renal function was evaluated by preoperative 99mTc-DMSA renal scan.

The metabolic abnormalities were addressed accordingly during follow-up. Patients with idiopathic hypercalciuria were treated with 50 mg/day thiazides titrated and patients with hypocitraturia were treated with potassium citrate with variable doses from 20 to 60 mEq/day depending on normal urinary citrate target and side effects. All patients underwent annual specialized medical consultation, serum creatinine evaluation, and ultrasonography (US) until the end of follow-up. Each new urinary stone found in US was confirmed by a computerized tomography. The occurrence of a new event related to lithiasis was defined as a new stone formation or stone elimination. The renal function was evaluated through chronic kidney disease epidemiology collaboration (CKD-EPI) equation^11^.

A multivariate logistic regression model was used to identify the urinary metabolic predictors of urolithiasis recurrence in the remaining kidney. The SPSS Advanced Statistics 24.0 program was used and the level of significance was defined as less than 5%.

## Results

Sixty-seven patients were included in this study (Table 1). Average follow-up was 71.6 ± 30.8 months in MAP group and 55.3 ± 25.4 months in CaOx group (*p* = 0.28).

**Table 1:** Descriptive analysis

		MAP (n=28)	CaOx (n=39)	p-value
Female		25 (89.2)	31 (79.4)	0.28
Age (y)		48.8 ± 14.9	51.8 ± 12.3	0.38
BMI - mean/SD (kg/m^2^)		26.47	27.51	0.77
Mean arterial pressure - mean/SD (mmHg)		9.5±2.1	9.8±1.7	0.6
Follow up - mean/SD (m)		71.6 ± 30.7	55.2 ± 25.3	0.28
Charlson >2		6 (21.4)	14 (35.9)	0.2
ASA	1	11 (39.3)	8 (20.5)	0.14
	2	12 (42.9)	28 (71.8)	
	3	4 (14.3)	2(5.1)	
	4	1 (3.6)	1 (2.6)	
Preoperative CKD-EPI	1	8 (28.6)	7 (17.9)	0.74
	2	11 (39.3)	19 (48.7)	
	3	9 (32.1)	13 (33.4)	
DMSA (affected kidney) %		7.16 ± 8.79	6.94 ± 8.62	0.93
Serum creatinine - mean/SD (mg/dL)		0.9 ± 0.2	1.0 ± 0.2	0.28
New event (yes)		5 (17.9)	9 (23.1)	0.6

CaOx: calcium oxalate; MAP: magnesium ammonium phosphate; BMI: body mass index; ASA: American Society of Anesthesiologists; CKD EPI: Chronic Kidney Disease Epidemiology Collaboration; DMSA: technetium-99m dimercaptosuccinic acid.

The metabolic evaluation mean time was 18.3 ± 12.7 months. The frequency of abnormalities in 24-hour urine samples was similar between groups (Table 2). In the MAP group, 71.4% of patients had at least one metabolic abnormality compared to 66.6% in the CaOx group (*p* = 0.67). Hypercalciuria occurred in 7.1 and 10.3% of patients in MAP and CaOx groups, respectively (*p* = 0.66), whereas hypocitraturia was present in 65.2 and 59.0% of patients from MAP and CaOx groups, respectively (*p* = 0.41). No difference in hypocitraturia rate was observed between 1 and 2-3 CKD-EPI grades (*p* = 0.45).

**Table 2:** Analysis of 24-hour urine composition

	MAP (n=28)	CaOx (n=39)	p-value
Volume - mean/SD (mL)	1813.4 ± 367.2	1782.4 ± 591.9	0.82
Hypercalciuria	7.1%	10.3%	0.66
Hyperoxaluria	0	0	1.00
Hypocitraturia	65.2%	59.0%	0.41
Hypernatriuria	13.0%	10.3%	0.52
Hyperuricosuria	0	5.1%	0.39

CaOx: calcium oxalate; MAP: magnesium ammonium phosphate.

No significant difference in new events between MAP and CaOx group (17.9 vs. 23.1%, respectively; *p* = 0.60) was found. The mean time to new event after nephrectomy was higher in the MAP group (66.8 ± 32.9 months vs. 50.3 ± 27.7 months, respectively; *p* = 0.04). Three patients from CaOx group and two from MAP group spontaneously passed stones during follow-up. Nine patients from CaOx group and five from MAP group formed new stones in the remained kidney. Stone analysis revealed calcium oxalate composition.

**Figure 1:**
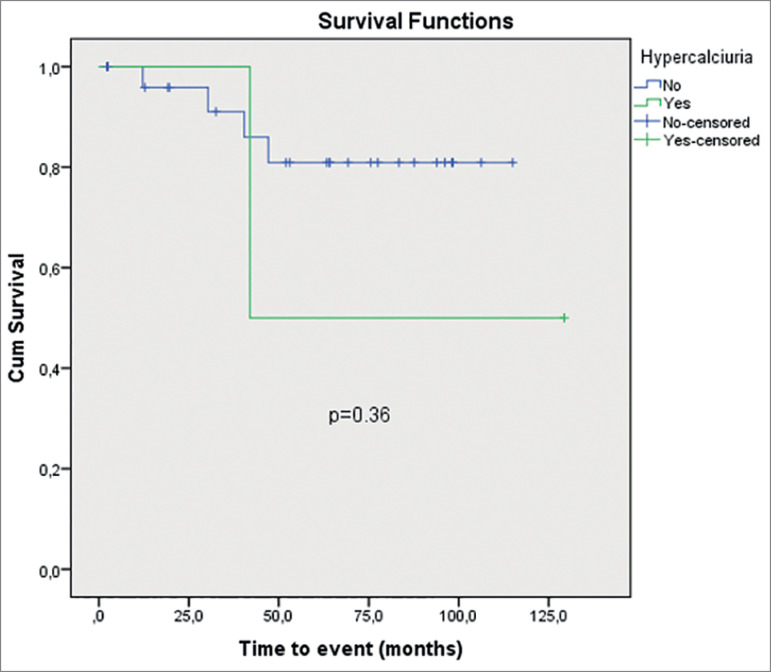
Time to new event in MAP group at hypercalciuria.

**Figure 2:**
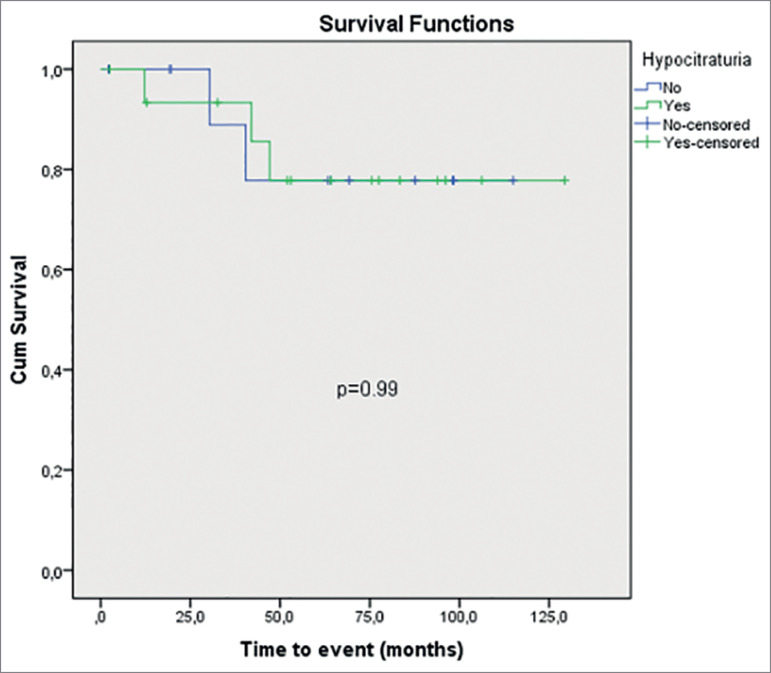
Time to new event in MAP group at hypocitraturia.

**Figure 3:**
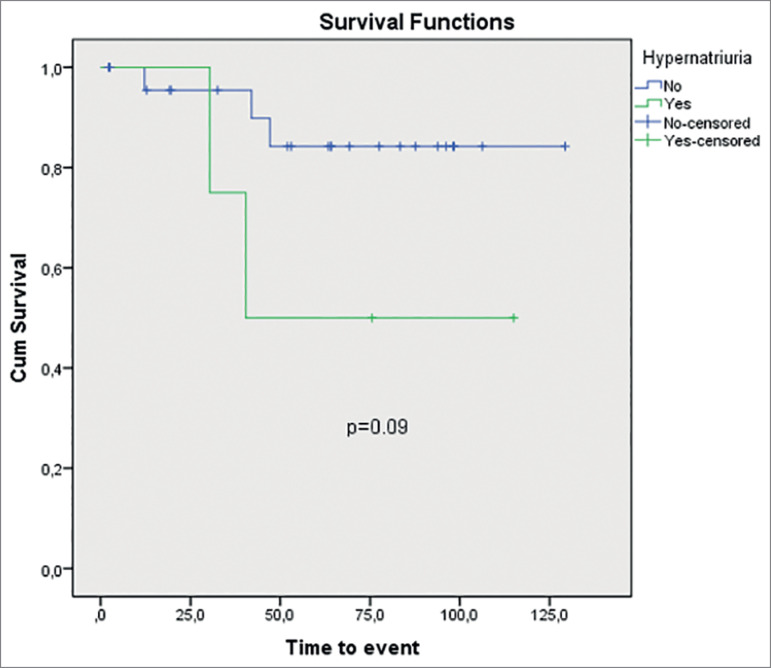
Time to new event in MAP group at hypernatriuria.

The risk for new event was not associated with the diagnosis of metabolic abnormalities in MAP group (*p* = 0.36) (Table 3). The actuarial curves of new event in MAP group with hypercalciuria, hypocitraturia, and hypernatriuria are shown in Figure 1, 2, and 3, respectively. The presence of urinary metabolic abnormalities did not influence the occurrence of new event in the MAP group (Table 4). Multivariate logistic regression of 24-hour urinary risk factors did not predict stone recurrence in the remaining kidney (Table 5).

**Table 3:** Association between metabolic abnormalities and new events in MAP group

	New event (R^2^)	p-value
Hypercalciuria	0.07	0.22
Hypocitraturia	0.009	0.66
Hypernatriuria	0.02	0.45

**Table 4:** Actuarial of new event versus metabolic disturbances in MAP group

	Time to new event (months)	p-value
Hypercalciuria (mg/24h) - mean ± SD	108.6 ± 8.2 [95%CI= 92.5-124.7]	0.36
Hypocitraturia (mg/24h) - mean ± SD	59.7±7.4 [95%CI= 63.1-92.0]	0.99
Hypernatriuria (mg/24h) - mean ± SD	113.1 ± 7.3 [IC95%= 98.7 - 127.6]	0.09

MAP: magnesium ammonium phosphate.

**Table 5:** Multivariate logistic regression for urolithiasis recurrence in remaining kidney

	OD	95% CI	p-value
Hypercalciuria	0.2	0.04-1.4	0.1
Hypocitraturia	2.0	0.5-7.5	0.2
Hypernatriuria	1.7	0.1-15.7	0.6

OD: odds ratio (for each increase of 1 unit).

## Discussion

We reported a high rate of 24-hour urine metabolic abnormalities in patients submitted to nephrectomy due to MAP stones. Stone recurrence rate was similar to CaOx stone formers (17.4 *vs*. 23.1%, *p*=0.373, respectively) in a long follow-up by annual ultrasound, confirmed by computerized tomography. We believe 24-hour urine analysis for patients who underwent nephrectomy by MAP stones is as important as for CaOx stones formers.

Nephrolithiasis is a disease with a high recurrence rate, resulting in decreased quality of life and renal function loss in the long term^12^. A recent retrospective study evaluating 2,200 first-time urinary stone formers found a recurrence rate of 11, 20, 31, and 39% after 2, 5, 10, and 15 years^13^. Thus, treatment focused on stone formation prevention is crucial to decrease morbidity and costs. In addition, urinary lithiasis may contribute to the worsening of renal function in patients undergoing nephrectomy^14^.

Urinary stone formers are more likely to have urinary metabolic abnormalities than the healthy population^15^. Although metabolic assessment has been performed mainly for recurrent stone formers^16^, Eisner et al. did not find differences in urinary metabolic abnormalities between first-time patients and patients with recurrent calculi. These authors suggested that metabolic evaluation should be offered even to patients with urinary stone for the first time^17^. Patients with infection stones are considered to be at high risk for recurrence and should undergo metabolic assessment, as recommended by the European Urology Association^15^.

The relationship between MAP stones and urinary infection is well established. These stones are formed in urine containing urease-producing bacteria, resulting in ammonia saturation and high urinary pH. The excess of ammonia is associated with phosphate and magnesium ions, forming MAP complexes^18^. The gold standard treatment for MAP stones is the complete elimination of calculi, because there is a chance of relapse due to bacteria stored in the calculi, even with urine sterilization through antibiotic treatment^19^. However, even with MAP stone eradication through surgical procedures such as extracorporeal lithotripsy, flexible ureteroscopy, and percutaneous nephrolithotripsy, and urine sterilization, several authors reported recurrence of nephrolithiasis (20 to 47%) associated with urinary risk factors^7^^,^ 14. Analyzing a small series, Lingeman et al. found metabolic abnormalities in 0.14% of patients with struvite stones^20^. The low rate of stone recurrence was also used to justify that the metabolic evaluation would be unnecessary in these patients. Silverman et al. reported 2.5% recurrence rate in 7-year follow-up of 40 patients with struvite stones^21^. Cicerello et al. showed hypercalciuria and hyperoxaluria in 10.5% (2/19) of patients evaluated with pure struvite stones^19^. On the other hand, several authors found a high rate of metabolic abnormalities in 24-hour urine and stone recurrence in patients with struvite stones^22^^-^ 24. In a recent series evaluating groups of patients with pure and combined struvite stones, the rate of metabolic abnormality was 57 and 81%, respectively^8^.

In the current study, hypocitraturia was found in 65.2% of patients over 3 months after been submitted to nephrectomy due to MAP stones. It is known that low levels of citrate due to metabolic deficiency may cause calcium precipitation^25^. In renal tubular acidosis at CKD onset, intracellular acidosis also leads to a higher proximal tubular reabsorption of citrate, resulting in significant hypocitraturia^26^. In our study, there was no difference in citraturia rate in patients with 1, 2, and 3 CKD grade (*p*=0.45). The high rate of hypocitraturia observed after nephrectomy might indicate that these patients are at risk of new stone formation due to metabolic cause and not only due to urinary infection. Also, we demonstrated that the treatment of hypocitraturia after the eradication of struvite stones equalizes stone recurrence rate to patients without hypocitraturia. Citrate is a known inhibitor of stone formation. Citrate reduces the availability of ionic calcium to interact with oxalate or phosphate in renal tubules^27^, helping the inhibitory effects of macromolecular modulators on calcium oxalate crystallization processes^28^. Also, it prevents crystal agglomeration and growth through its ability to bind to the crystal's surface and it prevents adhesion of calcium oxalate to renal epithelial cells^29^. However, the relatively low gastrointestinal tolerability of available alkali citrate preparations is the main limitation of its widespread usage. Jendle-Bengten et al., in a retrospective study, showed that only 62% of the patients adhered to potassium citrate treatment in the long term^30^.

Hypercalciuria is an important risk factor for urinary calculi, occurring in 35-65% of calcium stones formers^31^. We identified hypercalciuria in 7.1% of patients in the MAP group, while 10.3% in CaOx group presented this abnormality. The low rates of hypercalciuria in the present study may be associated to the high proportion of patients with variable degrees of impairment of renal function, 71.4% in MAP group and 82.1% in CaOx group. Measures such as adequate fluid and sodium intake in addition to the use of thiazides may reduce the urinary calcium excretion^32^, which may prevent formation and growth of apatite crystal, having a positive impact in preventing these stones.

The small sample size, chemical stone analysis, and the impossibility of analyzing the nature of all recurrent urinary stones are shortcomings and limitations of this study. Chemical analysis is not the gold standard to determine urinary stone composition. However, we tried to reduce this limitation by including only "pure" CaOx and MAP stones. Therefore, mix stone composition would not contaminate our sample. However, we cannot determine the precision of the chemical analysis for "pure" stones because the method itself has poor reliability.

In conclusion, our study highlights the need for a 24-hour urinary assessment even in pure MAP stone formers after the eradication of stones. Patients submitted to nephrectomy due to pure MAP stones have similar risk of 24-hour urinary abnormalities as their CaOx counterparts. Moreover, when these 24-hour urinary abnormalities are treated, the risk of new stone-related events are similar to patients without any metabolic abnormalities.
